# Synthesis of 2-alkylthio-*N*-(quinazolin-2-yl)benzenesulfonamide derivatives: anticancer activity, QSAR studies, and metabolic stability

**DOI:** 10.1007/s00706-018-2251-6

**Published:** 2018-07-13

**Authors:** Aneta Pogorzelska, Beata Żołnowska, Jarosław Sławiński, Anna Kawiak, Krzysztof Szafrański, Mariusz Belka, Tomasz Bączek

**Affiliations:** 1 Department of Organic Chemistry, Medical University of Gdańsk, Gdańsk, Poland; 2 Department of Biotechnology, Intercollegiate Faculty of Biotechnology, University of Gdańsk and Medical University of Gdańsk, Gdańsk, Poland; 3 Laboratory of Human Physiology, Medical University of Gdańsk, Gdańsk, Poland; 4 Department of Pharmaceutical Chemistry, Medical University of Gdańsk, Gdańsk, Poland

**Keywords:** 2-Alkylthiobenzenesulfonamide, Quinazoline, Anti-tumor agents, QSAR, Metabolic stability, ADMET

## Abstract

**Abstract:**

A new series of 2-alkylthio-*N*-(quinazolin-2-yl)benzenesulfonamide derivatives have been synthesized and evaluated in vitro for their antiproliferative activity by MTT assay against cancer cell lines HCT-116, MCF-7, and HeLa as well as the NCI-60 human tumor cell lines screen. In NCI screen, three compounds inhibited approximately 50% growth of RPMI-8226 and A549/ATCC cell lines. The mean of IC_50_ calculated in MTT assays for three tested cell lines was about 45 μM for four compounds. The QSAR allowed finding statistically significant OPLS models for HeLa cell line. Metabolic stability in vitro studies indicated favorable and unfavorable structural elements. The good metabolic stability, with *t*_1/2_ higher than 40 min, was observed for three derivatives, which together with their antiproliferative activity and good ADMET profile, makes them good leading structures for further research.

**Graphical abstract:**

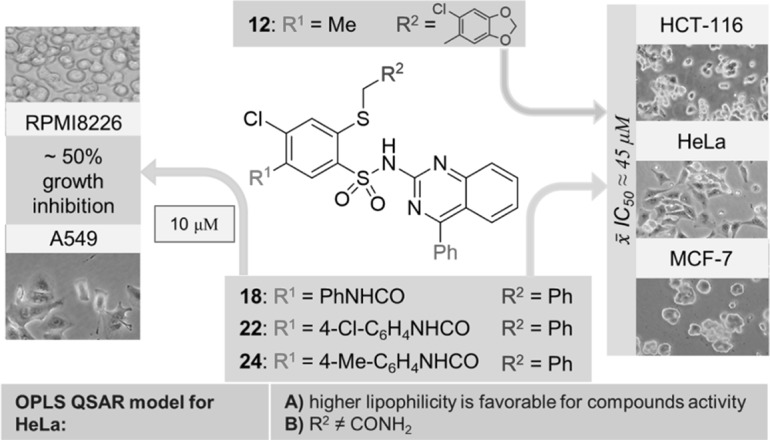

## Introduction

Cancer diseases are the second leading cause of death in developed countries and are expected to surpass heart diseases as the leading cause of death in the next few years [1]. Furthermore, finding new antineoplastic agents constitutes a great challenge for medicinal chemistry as their molecular mechanisms of action needed to be based on subtle differences in biochemical processes of healthy and cancerous cells.

**Fig. 1:**
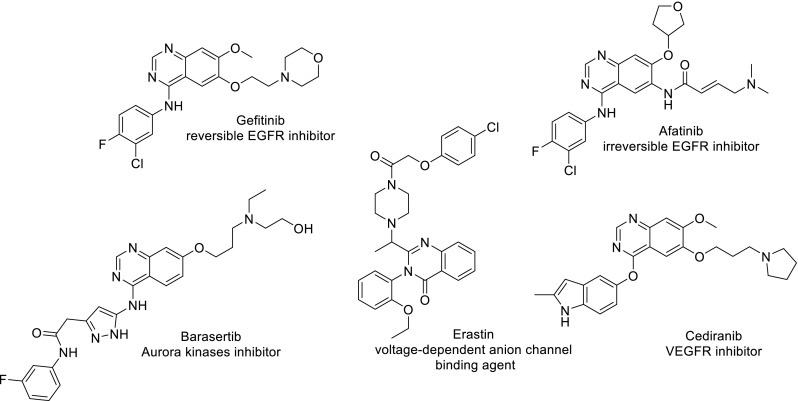
Anti-cancer quinazoline derivatives

Aryl sulfonamide derivatives are a group of compounds widely used in medicine, and show an interesting spectrum of anti-tumor activity [2, 3]. Among them, one of the most important and the largest is a group of *N*-arylsulfonamides in which the aryl/heteroaryl substituent is connected directly to the sulfonamide nitrogen atom [4–8]. Our team, carrying research on a multidirectional activity of various 2-mercaptobenzenosulfonamide (MBSA) derivatives, have found high anticancer potential in this class of compounds [9–12]. Thus, aiming to obtain a synergistic effect by combining MBSA scaffold and an aryl substituent with proven biological activity, we decided to synthesize and examined the anti-tumor properties of the 4-chloro-2-thio-*N*-(quinazolin-2-yl)benzenesulfonamides **9**–**24** (Scheme 1) in which the quinazoline ring is attached to the 2-mercaptobenzenesulfonamide core. The choice of quinazoline as *N*-aryl substituent was prompted by the fact that its derivatives are being examined for the enormous spectrum of biological activity [13–15], including anti-HIV, anti-inflammatory, anti-microbial, and anti-tumor activity as well as occurs in several approved drugs such as gefitinib, erlotinib, vandetanib, or raltitrexed [16]. The structures of some quinazoline-bearing compounds with anticancer activity are presented in Fig. 1.

**Figure:**
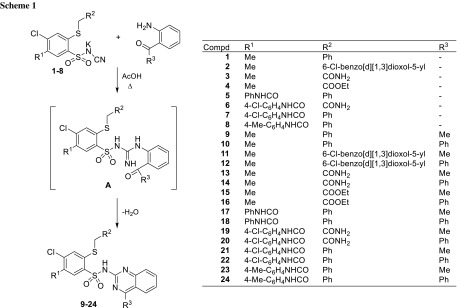

The mechanism of action of the most important clinically approved anticancer quinazolines is reversible (lapatinib, erlotinib, gefitinib) [17–20] or irreversible (afatinib) [17, 21] inhibition of EGFR tyrosine kinase receptors. Furthermore, in clinical and pre-clinical trials, there are several other quinazoline derivatives with unique anticancer molecular mechanisms such as cediranib—anti-angiogenic VEGFR inhibitor [22, 23], barasertib aurora kinases inhibitor [24, 25], or erastin altering mitochondrial voltage-dependent anion channels leading to induction of ferroptosis [26, 27]. Despite the facts about the anti-tumor activity of quinazolines and *N*-arylsulfonamides, there are only a few reports on the anti-tumor activity of *N*-(quinazolin)sulfonamide derivatives [28–30] against several human cancer cell lines, i.e., SCLC cell lines NCIH889, NCI-H1963, and NCI-H146, lung cancer cell line (A549), cervical (HeLa) cancer cell line, colorectal cell line (LoVo), and breast cancer cell line (MDA-MB-231). However, there is no such research on *N*-(quinazolin-2-yl)sulfonamides. Therefore, we hope that presented research on the synthesis and the in vitro anticancer activity of 2-alkylthio-4-chloro-*N*-(quinazolin-2-yl)benzenesulfonamide derivatives will provide new information and will lead to better understanding of the structure–activity relationships of *N*-(quinazolin)sulfonamides.

## Results and discussion

### Chemistry

As presented in Scheme 1, the desired 2-alkylthio-*N*-(quinazolin-2-yl)benzenesulfonamide derivatives **9**–**24** have been obtained by reacting the appropriate *N*-(2-alkylthio-4-chloro-5-methylbenzenesulfonyl)cyanamide potassium salts **1**–**8** with 2′-aminoacetophenone or 2-aminobenzophenone. The syntheses were carried out in glacial acetic acid by refluxing for 3.5–5 h and the reactions progression were monitored using TLC method. According to Scheme 1, at the initial step the *N*-(benzenesulfonyl)cyanamide potassium salts **1**–**8** react with amine group yielding an intermediate of type **A**, which is then cyclized to the desired final products.

The structures of new compounds **9**–**24** were confirmed by spectroscopic methods IR, ^1^H NMR, and ^13^C NMR as well as HRMS spectrometry and elemental analyses. IR spectra of compounds **9**–**24** showed absorption bands derived from NH group in the range 3396–3159 and 1670–1618 cm^−1^. The bands at range of 1379–1309 and 1165–1141 cm^−1^ were due to SO_2_ group. For derivatives **13** and **14** and **17**–**24,** the characteristic absorption bands for C=O amide groups from 1700 to 1641 cm^−1^ were observed. The ester C=O absorption bands in compounds **15** and **16** were detected at 1729 and 1723 cm^−1^, respectively.

The ^1^H NMR spectra of the series of *N*-(quinazolin-2-yl)benzenesulfonamides **9**–**24** revealed singlet signals at range 3.66–4.39 ppm for 2 protons of the methylthio group. The odd-numbered compounds gave singlet signals at around 2.5 ppm, which correspond to three protons from methyl group (R^3^ = Me). In turn, the spectra of compounds **17**–**24** having unsubstituted phenylcarbamoyl- or 4-substituted phenylcarbamoyl moiety, showed singlet signals in the range of 10.48–10.77 ppm attributable to NH proton of the amide function (CONH–Ar). Additionally, the ^1^H NMR spectra of all compounds showed singlet or broad singlet signal of the SO_2_NH proton in the downfield region *δ* = 12.50–13.75 ppm.

### Cytotoxic activity

All of the newly synthesized compounds **9**–**24** were evaluated for their effects on cell viability in three human cancer cell lines: HCT-116 (colon cancer), HeLa (cervical cancer), and MCF-7 (breast cancer). Cisplatin was used as reference drug. The results expressed as the concentration required for 50% inhibition of cell viability IC_50_ have been shown in Table 1. Among studied compounds, derivatives **12**, **18**, **22**, and **24** displayed the most potent cytotoxic effects against all tested cell lines. The mean IC_50_ values were 44.67 µM for **12**, **18**, and **22** while IC_50_ of **24** was 45.67 µM. On the other hand, compounds **13**–**15**, **19**, and **20** showed no antiproliferative effects. This suggests that amide group (R^2^ = CONH_2_) is undesirable, regardless of the kind of both R^1^ and R^3^, and aromatic ring in R^2^ position seem to be necessary for anticancer activity. Considering the activity of derivatives **9**–**12**, **15**, **17**, **18**, and **21**–**24**, it can be noticed that phenyl group as R^3^ bring an increase of cytotoxicity in comparison with methyl in place of R^3^. However, this impact seems to be the most important for compounds with the bulky group in R^1^ and generally, it is less for a set of derivatives with methyl group substituted in R^1^ (**9**–**12**).

**Table 1:** IC_50_ values for compounds **9**–**24**

Compd	IC_50_/µM			HaCaT
	HCT-116	HeLa	MCF-7	
**9**	61 ± 1	75 ± 5	100 ± 2	–
**10**	44 ± 1	61 ± 2	57 ± 1	–
**11**	46 ± 1	58 ± 2	57 ± 2	–
**12**	37 ± 1	46 ± 1	51 ± 2	64 ± 2
**13**	260 ± 5	320 ± 22	440 ± 4	–
**14**	85 ± 3	120 ± 1	155 ± 9	–
**15**	110 ± 2	230 ± 7	160 ± 6	–
**16**	62 ± 1	74 ± 4	105 ± 3	–
**17**	84 ± 3	72 ± 1	175 ± 10	–
**18**	42 ± 1	46 ± 2	46 ± 1	68 ± 2
**19**	100 ± 4	117 ± 5	105 ± 3	–
**20**	140 ± 4	110 ± 7	92 ± 1	–
**21**	54 ± 1	71 ± 2	115 ± 2	–
**22**	39 ± 1	42 ± 0.5	53 ± 1	59 ± 1
**23**	70 ± 1	75 ± 2	97 ± 2	–
**24**	41 ± 1	44 ± 3	52 ± 3	58 ± 1
Cisplatin	3.8 ± 0.2	2.2 ± 0.1	3.0 ± 0.1	

– Not tested

For the compounds with the strongest cytotoxicity (**12**, **18**, **22**, and **24**), an investigation of cytotoxic effect against non-carcinogenic cell line HaCaT was done. All compounds displayed promising selectivity toward cancer cells, especially HCT-116 and HeLa lines (Table 1). The activity against HCT-116 was 1.7-, 1.6-, 1.5-, and 1.4-fold higher when compared with HaCaT cell line, for compounds **12**, **18**, **22**, and **24**, respectively. In turn, the inhibition of the growth of HaCaT cells was 1.4-, 1.5-, 1.4-, and 1.3-fold weaker than HeLa cells, for compounds **12**, **18**, **22**, and **24**, respectively.

Aside from above, compounds **9**–**14** and **17**–**24** were also submitted to National Cancer Institute and evaluated for the cytotoxic effects toward 60 cell lines at a single dose of 10 μM (Table 2). These compounds exhibited preferential growth inhibition effects toward either leukemia or non-small cell lung cancer cell lines. As with MTT assays against HCT-116, HeLa and MCF-7, compounds **12**, **18**, **22**, and **24** showed the strongest antiproliferative effect while derivatives **13**, **14**, **19**, and **20** exhibited very weak cytotoxic activity. As it was summarized in Table 2, leukemia RPMI-8226 and NSCLC A549/ATCC were most sensitive to compounds **18**, **22**, and **24**. These derivatives in 10 μM concentration inhibited the growth of approximately 50% cells belonging to the above mention cell lines.

**Table 2:** The inhibition growth percent of selected NCI-60 cancer cells (IGP) at a single concentration of 10^−5^ M of compounds **9**–**14** and **17**–**24**

Panel	Cell line	IGP/% of compound													
		9	10	11	12	13	14	17	18	19	20	21	22	23	24
Leukemia	MOLT-4	12	25	20	12	11	9	9	36	3	5	7	52	5	21
	RPMI-8226	15	25	32	2	^a^	^a^	22	56	3	10	12	52	17	59
	SR	6	14	3	^a^	12	17	12	28	4	6	3	40	8	23
NSCLC	A549/ATCC	^a^	N	34	14	^a^	^a^	11	47	^a^	^a^	8	51	6	48
	EKVX	3	6	17	^a^	5	^a^	18	35	^a^	^a^	8	28	15	45
	HOP-92	3	17	N	N	^a^	^a^	20	26	8	4	8	34	^a^	26
	NCI-H522	9	^a^	19	16	4	4	12	25	8	11	20	35	^a^	28
Colon cancer	HCT-116	^a^	8	30	6	^a^	^a^	5	31	4	^a^	4	39	^a^	44
Melanoma	UACC-62	29	29	23	33	^a^	^a^	14	39	^a^	8	6	40	^a^	28
Renal cancer	UO-31	27	5	15	25	8	2	24	3	7	3	22	35	21	25
Prostate cancer	PC-3	2	N	23	14	^a^	^a^	3	24	4	2	8	44	18	49
Breast cancer	MCF-7	2	9	4	5	6	^a^	^a^	14	4	3	5	20	8	23

*N* not tested

^a^Growth percent ≥ 100%

### QSAR studies

QSAR analysis was performed to extract information regarding possible structure–activity relationship (SAR), especially to point out the most important parameters controlling pharmacological effects [31, 32].

The three-dimensional structure of the all studied compounds was built and optimized using the Gaussian software (Gaussian Inc.) [33] by density functional theory (DFT) method and B3LYP/6-31G(d) basis set. Subsequently, structures with optimized geometry were submitted to molecular descriptors calculation using Dragon 7.0 software (Talete, Milano, Italy) [34]. Among over 5200 descriptors only those easily interpretable were selected, namely constitutional indices, ring descriptors, functional group counts, atom-centered fragments, atom-type E-state indices, CATS 2D, 2D Atom Pairs, molecular properties and charge descriptors [35].

**Fig. 2:**
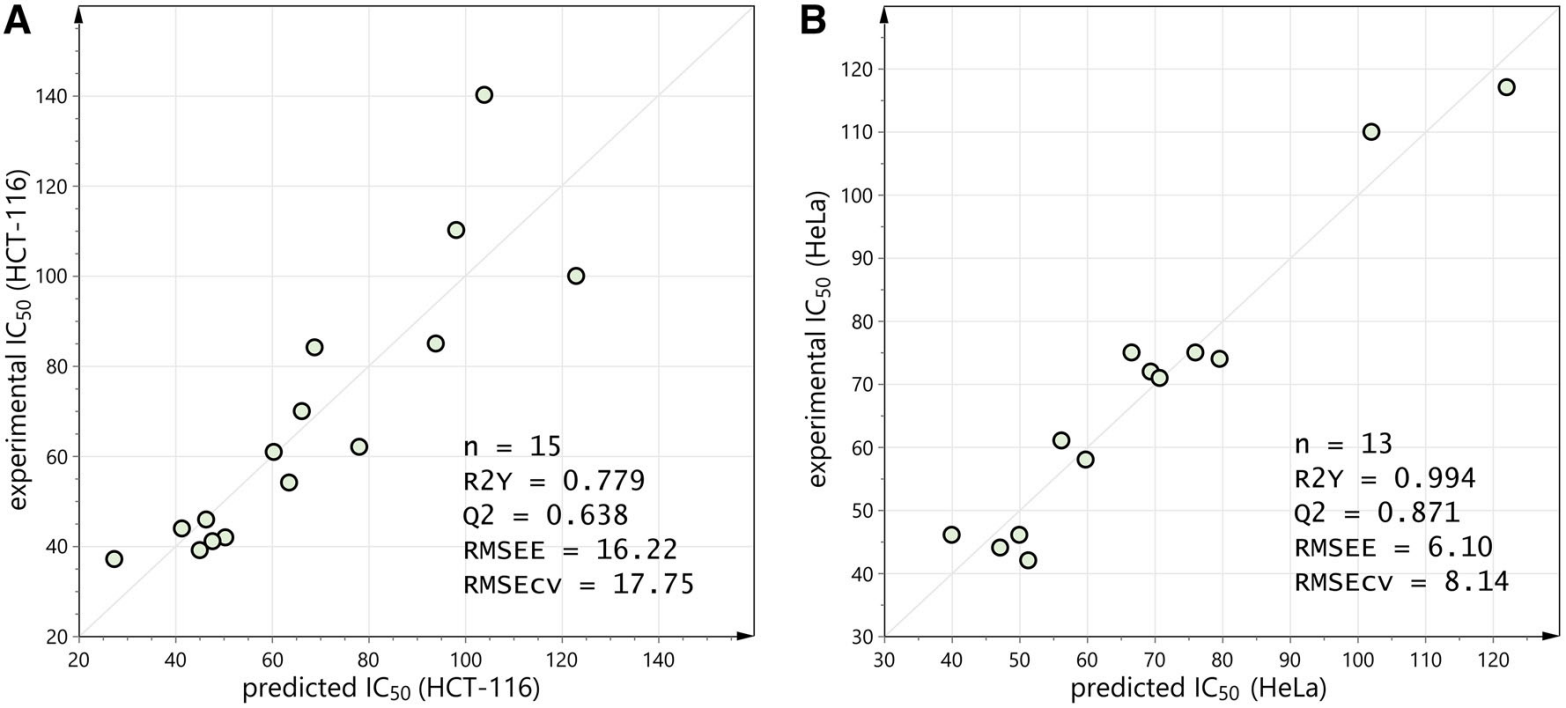
Plot of experimental versus predicted by OPLS model cytostatic activity of tested compounds towards HCT-116 (**a**) and HeLa (**b**) cell lines

Next, a multiple linear regression technique (MLR) was used to find QSAR equations correlating the cytotoxic activity expressed as IC_50_ with values of selected molecular descriptors used as independent variables. This basic approach led to statistically insignificant equations, so it was decided to apply multivariate PLS and OPLS techniques using SIMCA software [36, 37]. Such an approach enabled to achieve valuable information in one of our previous reports [38]. Before regression analysis compounds with outlying cytotoxicity values were excluded. For HeLa and HCT-116 cell lines, we established statistically significant OPLS models. Figure 2 shows the relation between observed and predicted IC_50_ values as well as some statistical parameters describing the models.

The model for HeLa cell line is able to describe over 99% of activity and predict over 87% of the variability in IC_50_ with cross-validated root mean squared error 8.14 µM.

The main advantage of OPLS is the possibility to point out relative influence of variables on the predictive model. The Variable Influence on Projection (VIP) values is used for such a comparison Table 3.

**Table 3:** List of molecular descriptors characterized by the highest VIP values in OPLS model built for cytostatic activity towards cervical cancer HeLa cell line

Descriptor	VIP	Full name of descriptor	Block of descriptors
ALOGP	4.83	Ghose–Crippen octanol–water partition coeff. (logP)	Molecular properties
ALOGP2	4.77	Squared Ghose–Crippen octanol–water partition coeff. (logP^2^)	Molecular properties
*N*%	4.70	Percentage of *N* atoms	Monstitutional indices
F04[C-S]	4.63	The frequency of C–S at topological distance 4	2D atom pairs
MCD	4.60	Molecular cyclized degree	Ring descriptors
nRCONH_2_	4.59	Number of primary amides (aliphatic)	Functional group counts

The two most important descriptors are logP and its square obtained from the Ghose–Crippen algorithm for ALOGP calculation [39]. Table 4 shows values of descriptors and uses shades of green color for easy visual interpretation. Obviously, higher lipophilicity is favorable for compounds activity. The third highest VIP value stands for *N*% descriptor that corresponds to the percent of *N* atoms in relation to all atoms in the molecule. It seems to be reversely proportional to logP as *N*-containing functional groups usually increase the polarity of a molecule. A more detailed analysis indicates that *N*% values in case of the present group of compounds relate specifically to unsubstituted amide in position R^2^ (compounds **19** and **20**). The presence of this functional group clearly changes values of nRCONH_2_ but also F04[C-S] into the less favorable level. MCD, the last among the most important descriptors, calculates molecular cyclized degree as a ratio between number of atoms belonging to any ring system and all atoms in the molecule [40]. There is an evidence that higher percent of the ring system is preferable for higher cytotoxic activity in this group of compounds.

**Table 4:**
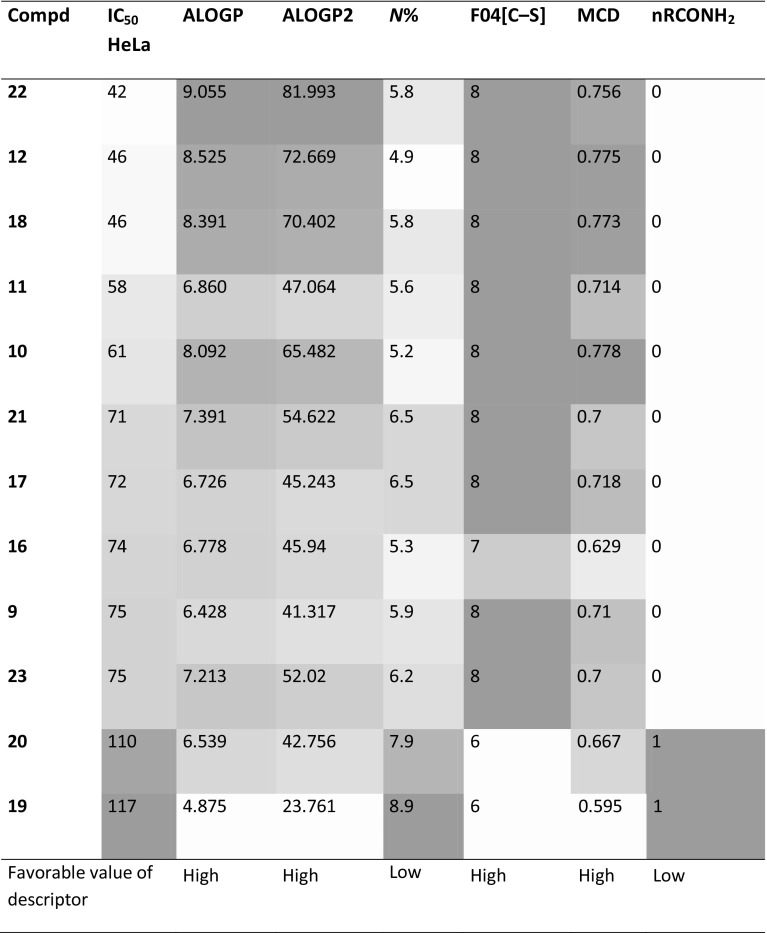
Values of descriptors selected as the most influential for OPLS model describing IC_50_ against HeLa cell line

Table is sorted with descending activity of compounds. Color intensity reflects descriptor values—the more intense color, the higher value

### Metabolic stability

**Fig. 3:**
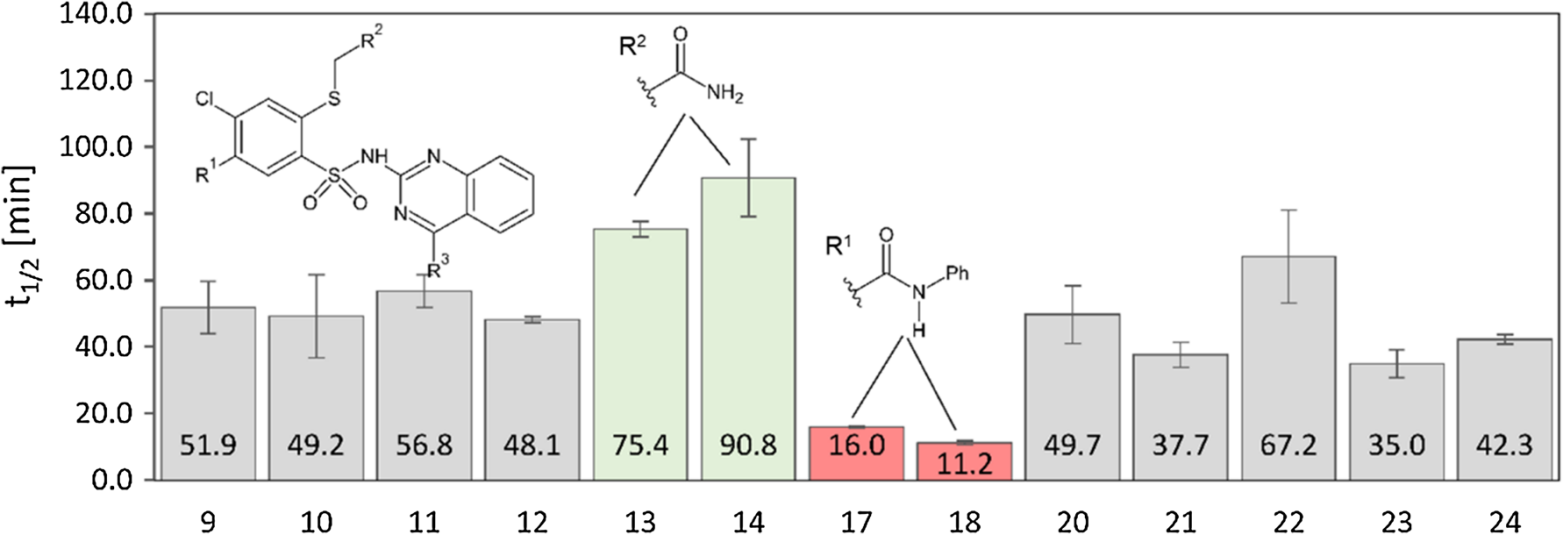
The in vitro metabolic half-life values, obtained in the presence of human liver microsomes and NADPH

Selected derivatives were submitted to metabolic stability study to assess their ability to remain unchanged in the presence of human metabolic enzymes. Human liver microsomes were used as they are a rich source of all common CYP isoenzymes. In vitro incubations were performed in the presence of NADPH as a cofactor and the disappearance of a derivative was followed using LC–MS technique. The in vitro metabolic half-life values are gathered in Fig. 3.

The selected group of derivatives varies significantly in the in vitro metabolic half-life values. The most stable and the least stable compounds are marked green and red, respectively (Fig. 3). High metabolic stability is a desirable property. Derivatives **13** and **14** are characterized by the best *t*_1/2_: 75.4 and 90.8 min, respectively. Interestingly, they both possess unsubstituted amide moiety in R^2^ position. Possibly, the amide is less reactive against metabolic enzymes than phenyl (**9** and **10**) or 6-chloro-2*H*-1,3-benzodioxole (**11** and **12**) substituents. On the other hand, compounds **17** and **18** are the least stable with half-life values 16.0 and 11.2 min, respectively. These derivatives also share the same chemical group-*N*-phenyl substituted amide in R^1^ position. The results show that additional substituent (either 4-chloro or 4-methyl) in R^1^ enable to increase metabolic stability twofold (**21** and **23** versus **17**) to four- to sixfold (**20**, **22**, **24** versus **18**). Regarding R^3^ position, there is no clear difference between methyl and phenyl substituents.

The most informative approach is to compare metabolic stability within one chemical group of derivatives. This way, we can assess which derivative is the most promising in particular group of new compounds. The comparison between different series of compounds is difficult due to the lack of standard experiment, which is widely used worldwide. For this reason, it is more advisable to compare metabolic stability between our previous reports, because we have used the same test to determine metabolic stability and calculate in vitro metabolic half-life. Our report [38] concerning sulfonamide derivatives with 1,3,4-oxadiazole ring revealed *t*_1/2_ between ca 5 and over 60 min, whereas another study [41] showed metabolic stability in a range 13–38 min. In this report, the most stable compounds **13** and **14** are thus one of the most stable among all tested sulfonamide derivatives. In the presented research, the substituent R^2^, amide group, is replaced by phenyl, ethyl ester, or 6-Cl-benzo[*d*] [1, 3] dioxol-5-yl substituent. The amide group is reported as a stable one. It is not vulnerable to oxidation mediated by CYP enzymes. However, substituted amides can be cleaved by enzymatic hydrolysis [42]. On the other hand, phenyl (as well as benzodioxole) ring can be easily hydroxylated in I phase metabolism [43]. Our findings confirm previous reports and show that incorporation of unsubstituted amide is beneficial for metabolic stability.

### ADMET in silico prediction

Based on the cytotoxicity studies, the compounds **12**, **18**, **22**, and **24** were selected for in silico predictions of absorption, distribution, metabolism, elimination, and toxicity (ADMET) processes. The ADMET properties of **12**, **18**, **22**, and **24** are detailed in Table 5.

**Table 5:** In silico ADMET parameters of compounds **12**, **18**, **22**, and **24** predicted by pkCSM approach [44]

Descriptor	Predicted value			
	12	18	22	24
Absorption				
logPapp	0.911	− 0.167	0.555	0.563
P-gp substrate	+	+	+	+
P-gp I inhibitor	+	+	+	+
P-gp II inhibitor	+	+	+	+
Distribution				
logVDss (human)	− 0.946	− 1.209	− 0.92	− 0.929
log BB	− 1.049	− 0.578	− 0.471	− 0.296
Metabolism				
CYP2D6 substrate	−	−	−	−
CYP3A4 substrate	+	+	+	+
CYP1A2 inhibitor	−	−	−	−
CYP2C19 inhibitor	+	−	+	+
CYP2C9 inhibitor	+	−	−	−
CYP2D6 inhibitor	−	−	−	−
CYP3A4 inhibitor	+	−	−	−
Toxicity				
Max. tolerated dose (log mg/kg/day)	0.426	0.436	0.443	0.444
hERG I inhibitor	−	−	−	−
hERG II inhibitor	+	+	+	+

In a drug discovery process, the Lipinski’s rule is the standard for the development of orally available drugs. However, an experience in drug development shows that highly promising drug candidate is often not accepted for further research because it did not comply the rule-of-five. Furthermore, the Lipinski’s rule seems to overemphasize since only 51% of all FDA-approved drugs comply with this protocol [45]. In the literature, there is a lot of in silico models to predict if drug candidate will be orally bioavailable. The Caco-2 monolayer is an in vitro model of the human intestinal mucosa. It is widely used to predict the absorption of orally administrated drugs. A compound has a high Caco-2 permeability if Papp > 8 × 10^−6^ cm/s (logPapp > 0.9). Although none of the studied compounds do comply with Lipinski’s rule, results in Table 5 showed that compound **12** is considered to have a high Caco-2 permeability. Furthermore, the results of the calculated property for absorption of compounds **23** and **24** revealed that these can be absorbed throughout the intestine but to a lower degree than **12**.

A distribution of compounds **12**, **18**, **22**, and **24** were determined as free parameters, namely volume of distribution (logVDss) and blood–brain barrier permeability (logBB). The VDss defines the drug distribution between plasma and the rest of the body. It is considered low if below 0.71 L/kg (log VDss < − 0.15) and high if above 2.81 L/kg (log VDss > 0.45). A compound is able to readily cross the blood–brain barrier if logBB > 0.3 while a molecule with logBB < − 1 is poorly distributed to the brain. The VDss of all studied derivatives was lower than − 0.15. A low volume of distribution indicates that there is a low probability of compounds accumulation in body tissues. On the other hand, the logBB was lower than − 1 only for compound **12**. However, derivatives **18** and **22** with logBB − 0.578 and − 0.471, respectively, seem to display limited brain penetration. The highest logBB = − 0.296 was observed for **24** but still, this is a low value. The obtained logBB values suggest that compounds display rather a low brain toxicity, if at all.

Compounds transport and metabolism were expressed as a modulation of the activity of P-glycoprotein (P-gp) and various cytochrome P450 enzymes. Modulation of P-glycoprotein transport is essential for compounds pharmacokinetic properties. A number of drugs, including drugs in cancer chemotherapy, are substrates of P-gp transporter [46]. The data in Table 5 indicate that all compounds are potential P-gp substrates. On the other hand, all of them are also P-gp inhibitors. The inhibition of P-gp activity is considered as a strategy in cancer therapy to avoid P-gp-mediated drug resistance [47]. However, an efficient P-gp inhibitor should not inhibit the activity of cytochrome P450 enzymes involved in drug metabolism, particularly CYP3A. The simultaneous inhibition of both P-gp transport and CYP3A metabolism may result in an increase plasma drug concentration and leads to higher toxicity [48]. The obtained predictions indicate that only compound **12** could potentially inhibit the activity of both P-gp and CYP3A4. Derivatives **18**, **22**, and **24** were not considered to have the capability for inhibiting CYP3A4. On the other hand, compounds **18**, **22**, and **24** are substrates of P-gp and CYP3A4. Owing to the complementary function of these proteins [46], this may result in a greater than expected reduction in systematic exposure to **18**, **22**, and **24**. Furthermore, these effects may allow for the maintenance of drug elimination when either CYP3A4 or P-gp activity is inhibited [46].

To consider the compounds toxicity, maximum tolerated dose and hERG inhibition were calculated in silico. The maximum dose of all compounds is significantly higher than their cytotoxic concentration. On the other hand, the discussed derivatives, as a considered hERG inhibitors, may cause drug-induced (acquired) QT interval prolongation associated with an increase in the incidence of sudden unexplained deaths. The hERG inhibition is considered as the main cause of it. However, the predicted ADMET properties indicated that only hERG II may be inhibited by 2-alkylthio-*N*-(quinazolin-2-yl)benzenesulfonamide derivatives. Thus, further studies with in vitro models are needed for compounds safety related with hERG inhibition.

## Conclusion

We have developed methods for the synthesis of novel series of 2-alkylthio-*N*-(quinazolin-2-yl)benzenesulfonamide derivatives. The compounds were tested in vitro by MTT assay for their cytotoxic activity against three cancer cell lines: colon HCT-116, cervical HeLa, and breast MCF-7. Furthermore, compounds **9**–**14** and **17**–**24** were evaluated by NCI as potential growth inhibitors of 60 human cancer cell lines. We have found that the studied compounds display moderate cytotoxic activity and the best antiproliferative effect was observed for compounds **18**, **22**, and **24** which showed good results in MTT assays as well as NCI studies. Aside from HCT-116, HeLa, and MCF-7, noticeable growth inhibition of RPMI-8226 and A549/ATCC were also observed. Quite good results of MTT assays were also noticed for compound **12**. The mean IC_50_ value against three tested cell lines was 44.67 µM (identical with IC_50_ obtained for **18** and **22**). What important, the activity of compounds **12**, **18**, **22**, and **24** was lower against non-cancerogenic HaCaT cell line than susceptible cancer cells. Structure–activity relationship revealed that amide group (R^2^ = CONH_2_) is undesirable, while aromatic ring in R^2^ position is important for anticancer effect. Furthermore, phenyl as R^3^ is more favorable than methyl and this impact is probably stronger in compounds with the bulky group in R^1^.

QSAR studies showed that higher lipophilicity is important for better compound activity against HeLa cell line. Furthermore, unsubstituted amide in position R^2^ is inadvisable for HeLa growth inhibition.

All but two (**17**, **18**) of the 2-alkylthio-*N*-(quinazolin-2-yl)benzenesulfonamide derivatives displayed good metabolic stability with *t*_1/2_ in the range of 35–90.8 min. Compounds **12**, **22**, and **24** were characterized by *t*_1/2_ higher than 40 min, which together with the outstanding activity, makes them good leaders for further research.

The predicted in silico ADMET properties of compounds **12**, **18**, **22**, and **24** indicated that new derivatives display rather low probability of side effects. However, further studies with in vitro models are needed to determine their complete safety profile.

## Experimental

Melting points were measured using Boethius PHMK apparatus. IR spectra were measured on Thermo Mattson Satellite FTIR spectrometer in KBr pellets; an absorption range was 400–4000 cm^−1^. ^1^H NMR and ^13^C NMR spectra were recorded on a Varian Gemini 200 apparatus or Varian Unity Plus 500 apparatus. Chemical shifts are expressed at *δ* values relative to Me_4_Si (TMS) as an internal standard. The apparent resonance multiplicity is described as: s (singlet), d (doublet), dd (doublet of doublets), t (triplet), m (multiplet), and br (broad) signal. The addition of equimolar TFA was necessary to obtain ^13^C NMR spectra. Due to a poor solubility of compounds **21** and **23**, the obtained ^13^C NMR spectra were not sufficient. HRMS analyses were performed on a TripleTOF 5600 + System (AB SCIEX, USA) in positive ion mode. Elemental analyses were performed on PerkinElmer 2400 Series II CHN Elemental Analyzer and the results were within ± 0.4% of the theoretical values. Thin-layer chromatography (TLC) was performed on Merck Kieselgel 60 F254 plates and visualized with UV. The commercially unavailable monopotassium salts were obtained according to the following methods described previously: **1**, **4** [49], **2** [50], **3**–**5**, **8** [51], and **7** [52].

### *N*-[4-Chloro-5-(4-chlorophenylcarbamoyl)-2-carbamoylthiobenzenesulfonyl]cyanamide potassium salt (6, C_16_H_11_Cl_2_KN_4_O_4_S_2_)

The mixture of 4.020 g 3-amino-6-chloro-*N*-(4-chlorophenyl)-1,4,2-benzodithiazine-7-carboxamide 1,1-dioxide (10 mmol), 1.026 g 2-chloroacetamide (11 mmol), and 8.26 g anhydrous K_2_CO_3_ (59.8 mmol) in 67 cm^3^ dry tetrahydrofuran was stirred for 7.5 h at reflux. After cooling the solid was filtered off, dried, treated with 58 cm^3^ water and stirred for 20 min. Crude product was filtered off and crystallized from ethanol, giving 3.460 g (70%) **6**. M.p.: 245–248 °C (dec.); ^1^H NMR (500 MHz, DMSO-*d*_*6*_): *δ* = 3.76 (s, 2H, CH_2_), 7.29 (s, 1H, NH_2_), 7.41 (d, 2H, arom), 7.64 (s, 1H, NH_2_), 7.65 (s, 1H, H-3), 7.72 (d, 2H, arom), 7.86 (s, 1H, H-6), 10.77 (s, 1H, NH) ppm; IR (KBr): $\bar{\nu }$ = 3462, 3357 (NH), 2923, 2853 (CH), 2176 (C≡N), 1671 (NH def), 1314, 1143 (SO_2_) cm^−1^.

### General procedure for the preparation of 2-alkylthio-*N*-(4-R^3^-quinazolin-2-yl)benzenesulfonamides 9–24

A mixture of the appropriate *N*-(benzenesulfonyl)cyanamide potassium salts **1**–**8** (2 mmol) and 2′-aminoacetophenone or 2-aminobenzophenone (2.2 mmol) in 6 cm^3^ glacial acetic acid was refluxed with stirring for 3.5–5 h. Then the mixture was cooled in an ice bath. The solid was collected by filtration, washed with glacial acetic acid (2 × 1 cm^3^) and dried. The final products **9**–**24** were purified as described below.

#### 2-Benzylthio-4-chloro-5-methyl-*N*-(4-methylquinazolin-2-yl)benzenesulfonamide (9, C_23_H_20_ClN_3_O_2_S_2_)

Starting from 0.780 g **1** to 0.299 g 2′-aminoacetophenone for 5 h, the crude product was obtained. Crystallization from 10 cm^3^ ethanol gave 0.400 g (43%) **9**. M.p.: 183–186 °C; TLC: *R*_f_ = 0.75 (benzene-EtOH 4:1); ^1^H NMR (200 MHz, DMSO-*d*_*6*_): *δ* = 2.39 (s, 3H, CH_3_), 2.58 (s, 3H, CH_3_), 4.25 (s, 2H, CH_2_), 7.04–7.06 (m, 3H, arom), 7.25–7.27 (m, 2H, arom), 7.40–7.50 (m, 3H, arom), 7.82–7.89 (t, 1H, arom), 8.02–8.12 (m, 2H, arom), 13.30 (br s, 1H, SO_2_NH) ppm; ^13^C NMR (125 MHz, DMSO-*d*_*6*_/TFA): *δ* = 19.1, 22.1, 36.4, 118.3, 120.3, 124.9, 127.0, 127.3, 127.4, 128.5, 128.6, 129.3, 131.7, 133.9, 135.7, 136.1, 136.6, 137.2, 138.8, 143.7, 153.1 ppm; IR (KBr): $\bar{\nu }$ = 3256 (NH), 1623 (NH def), 1580, 1524 (C=C, C=N), 1379, 1141 (SO_2_) cm^−1^; HRMS (ESI-TOF): *m/z* calcd. for C_23_H_20_ClN_3_O_2_S_2_ ([M+H]^+^) 470.0764, found 470.0764.

#### 2-Benzylthio-4-chloro-5-methyl-*N*-(4-phenylquinazolin-2-yl)benzenesulfonamide (10, C_28_H_22_ClN_3_O_2_S_2_)

Starting from 0.780 g **1** to 0.430 g 2-aminobenzophenone for 3.5 h, the crude product was obtained. Extraction with 7 cm^3^ boiling ethanol gave 0.590 g (55%) **10**. M.p.: 192–198 °C; TLC: *R*_f_ = 0.75 (benzene-EtOH 4:1); ^1^H NMR (200 MHz, DMSO-*d*_*6*_): *δ* = 2.20 (s, 3H, CH_3_), 4.26 (s, 2H, CH_2_), 6.86–6.87 (m, 3H, arom), 7.11–7.30 (m, 4H, arom), 7.42–7.53 (m, 3H, arom), 7.59–7.65 (m, 3H, arom), 7.82–7.97 (m, 3H, arom), 13.50 (br s, 1H, SO_2_NH) ppm; ^13^C NMR (125 MHz, DMSO-*d*_*6*_/TFA): *δ* = 19.1, 36.3, 117.2, 120.8, 125.3, 127.1, 127.9, 128.2, 128.4, 128.6, 129.1, 130.1, 131.1, 132.1, 133.6, 135.0, 135.8, 136.3, 136.7, 137.1, 139.4, 145.8, 153.1 ppm; IR (KBr): $\bar{\nu }$ = 3238 (NH), 2922 (CH), 1620 (NH def), 1582, 1567 (C=C, C=N), 1358, 1138 (SO_2_) cm^−1^; HRMS (ESI-TOF): *m/z* calcd. for C_28_H_22_ClN_3_O_2_S_2_ ([M+H]^+^) 532.0920, found 532.0910.

#### 4-Chloro-2-(6-chlorobenzo[1,3]dioxol-5-ylmethylthio)-5-methyl-*N*-(4-methylquinazolin-2-yl)benzenesulfonamide (11, C_24_H_19_Cl_2_N_3_O_4_S_2_)

Starting from 0.940 g **2** to 0.299 g 2′-aminoacetophenone for 5 h, the crude product was obtained. Crystallization from 40 cm^3^ acetonitrile gave 0.470 g (43%) **11**. M.p.: 198–200 °C; TLC: *R*_f_ = 0.78 (benzene-EtOH 4:1); ^1^H NMR (200 MHz, DMSO-*d*_*6*_): *δ* = 2.41 (s, 3H, CH_3_), 2.58 (s, 3H, CH_3_), 4.19 (s, 2H, SCH_2_), 5.90 (s, 2H, OCH_2_O), 6.83 (s, 1H, arom), 6.92 (s, 1H, arom), 7.42–7.48 (m, 3H, arom), 7.81–7.85 (t, 1H, arom), 8.01–8.05 (m, 2H, arom), 13.3 (br s, 1H, SO_2_NH) ppm; ^13^C NMR (50 MHz, DMSO-*d*_*6*_): *δ *= 19.19, 22.21, 34.65, 102.23, 109.63, 110.46, 124.78, 125.36, 126.83, 126.96, 127.71, 131.91, 135.22, 146.65, 147.61, 152.90 ppm; IR (KBr): $\bar{\nu }$ = 3279 (NH), 2920 (CH), 1618 (NH def), 1582, 1475 (C=C, C=N), 1363, 1165 (SO_2_) cm^−1^; HRMS (ESI-TOF): *m/z* calcd. for C_24_H_19_Cl_2_N_3_O_4_S_2_ ([M+H]^+^) 548.0272, found 548.0268.

#### 4-Chloro-2-(6-chlorobenzo[d][1,3]dioxol-5-ylmethylthio)-5-methyl-*N*-(4-phenylquinazolin-2-yl)benzenesulfonamide (12, C_29_H_21_Cl_2_N_3_O_4_S_2_)

Starting from 0.940 g **2** to 0.430 g 2-aminobenzophenone for 5 h, the crude product was obtained. Crystallization from 34 cm^3^ acetonitrile gave 0.500 g (43%) **12**. M.p.: 112–116 °C; TLC: *R*_f_ = 0.77 (benzene-EtOH 4:1); ^1^H NMR (200 MHz, DMSO-*d*_*6*_): *δ* = 2.23 (s, 3H, CH_3_), 4.21 (s, 2H, SCH_2_), 5.64 (s, 2H, OCH_2_O), 6.30 (br s, 1H), 6.89 (s, 1H, arom), 7.05–7.10 (m, 2H, arom), 7.40–7.51 (m, 3H, arom), 7.62 (m, 3H, arom), 7.79–7.96 (m, 3H, arom), 13.5 (br s, 1H, SO_2_NH) ppm; ^13^C NMR (125 MHz, DMSO-*d*_*6*_/TFA): *δ* = 19.0, 34.6, 102.0, 109.5, 110.6, 116.8, 120.1, 125.0, 125.3, 127.2, 128.4, 128.5, 128.6, 130.1, 131.1, 132.6, 133.4, 134.2, 135.6, 136.2, 137.0, 140.5, 145.2, 146.5, 147.4, 152.9 ppm; IR (KBr): $\bar{\nu }$ = 3239 (NH), 2919 (CH), 1619 (NH def), 1582, 1570, 1478 (C=C, C=N), 1357, 1141 (SO_2_) cm^−1^; HRMS (ESI-TOF): *m/z* calcd. for C_29_H_21_Cl_2_N_3_O_4_S_2_ ([M+H]^+^) 610.0429, found 610.0219.

#### 2-Carbamoylmethylthio-4-chloro-5-methyl-*N*-(4-methylquinazolin-2-yl)benzenesulfonamide (13, C_18_H_17_ClN_4_O_3_S_2_)

Starting from 0.720 g **3** to 0.299 g 2′-aminoacetophenone for 5 h, the crude product was obtained. Crystallization from 50 cm^3^ ethanol gave 0.360 g (40%) **13**. M.p.: 232–235 °C (dec); TLC: *R*_f_ = 0.43 (benzene-EtOH 4:1); ^1^H NMR (200 MHz, DMSO-*d*_*6*_): *δ* = 2.40 (s, 3H, CH_3_), 2.67 (s, 3H, CH_3_), 3.66 (s, 2H, CH_2_), 7.20 (s, 1H, CONH_A_), 7.39–7.63 (m, 4H, arom, CONH_B_), 7.81–7.88 (t, 1H, arom), 8.03–8.07 (m, 2H, arom), 13.35 (br s, 1H, SO_2_NH) ppm; ^13^C NMR (125 MHz, DMSO-*d*_*6*_/TFA): *δ* = 19.1, 22.3, 36.6, 118.3, 120.2, 125.0, 127.0, 127.2, 131.8, 133.9, 136.1, 136.2, 137.3, 138.5, 143.6, 153.3, 170.0 ppm; IR (KBr): $\bar{\nu }$ = 3393, 3176 (NH), 2918 (CH), 1659 (CO), 1631 (NH def), 1587, 1524, 1493 (C=C, C=N), 1362, 1145 (SO_2_) cm^−1^; HRMS (ESI-TOF): *m/z* calcd. for C_18_H_17_ClN_4_O_3_S_2_ ([M+H]^+^) 437.0509, found 437.0516.

#### 2-Carbamoylmethylthio-4-chloro-5-methyl-*N*-(4-phenylquinazolin-2-yl)benzenesulfonamide (14, C_23_H_19_ClN_4_O_3_S_2_)

Starting from 0.720 g **3** to 0.430 g 2-aminobenzophenone for 5 h, the crude product was obtained. Crystallization from 59 cm^3^ DMSO/methanol (v/v 29:30) gave 0.350 g (36%) **14**. M.p.: 275–279 °C (dec.); TLC: *R*_f_ = 0.54 (benzene-EtOH 4:1); ^1^H NMR (200 MHz, DMSO-*d*_*6*_): *δ* = 2.22 (s, 3H, CH_3_), 3.67 (s, 2H, CH_2_), 7.19 (s, 1H, CONH_A_), 7.40–7.66 (m, 9H, arom, CONH_B_), 7.85–7.99 (m, 3H, arom), 13.58 (br s, 1H, SO_2_NH) ppm; ^13^C NMR (125 MHz, DMSO-*d*_*6*_/TFA): *δ* = 18.8, 36.5, 117.2, 120.7, 125.1, 127.5, 128.2, 128.6, 130.1, 131.1, 132.2, 133.8, 135.1, 135.5, 136.0, 136.2, 137.4, 138.8, 145.7, 153.3, 170.0 ppm; IR (KBr): $\bar{\nu }$ = 3441 (NH), 2921 (CH), 1650 (CO), 1629 (NH def), 1584, 1453 (C=C, C=N), 1363, 1139 (SO_2_) cm^−1^; HRMS (ESI-TOF): *m/z* calcd. for C_23_H_19_ClN_4_O_3_S_2_ ([M+H]^+^) 499.0665, found 499.0518.

#### 4-Chloro-2-(2-ethoxy-2-oxoethylthio)-5-methyl-*N*-(4-methylquinazolin-2-yl)benzenesulfonamide (15, C_20_H_20_ClN_3_O_4_S_2_)

Starting from 0.775 g **4** to 0.299 g 2′-aminoacetophenone for 5 h, the reaction mixture was evaporated under the diminished pressure and the residue was crystallized from 2 cm^3^ acetonitrile. The crude product (0.640 g, 69%) was filtered and dried. Crystallization from 31 cm^3^ ethanol gave 0.280 g (30%) **15**. M.p.: 192–194 °C; TLC: *R*_f_ = 0.71 (benzene-EtOH 4:1); ^1^H NMR (200 MHz, DMSO-*d*_*6*_): *δ* = 0.95–1.09 (m, 3H, CH_3_), 2.41 (s, 3H, CH_3_), 2.65 (s, 3H, CH_3_), 3.87–3.98 (m, 4H, CH_2_, SCH_2_), 7.32–7.52 (m, 3H, arom), 7.84 (t, 1H, arom), 8.04 (d, 1H, arom), 8.14 (s, 1H, arom), 13.30 (br s, 1H, SO_2_NH) ppm; ^13^C NMR (125 MHz, DMSO-*d*_*6*_/TFA): *δ* = 13.8, 18.9, 22.0, 34.5, 61.2, 118.2, 120.0, 124.8, 126.8, 127.4, 132.1, 134.0, 134.6, 136.0, 137.2, 143.4, 153.2, 169.2 ppm; IR (KBr): $\bar{\nu }$ = 3258 (NH), 2976, 2918 (CH), 1729 (C=O), 1623 (NH def), 1579, 1524, 1497 (C=C, C=N), 1358, 1140 (SO_2_) cm^−1^; HRMS (ESI-TOF): *m/z* calcd. for C_20_H_20_ClN_3_O_4_S_2_ ([M+H]^+^) 466.0662, found 466.0517.

#### 4-Chloro-2-(2-ethoxy-2-oxoethylthio)-5-methyl-*N*-(4-phenyl-quinazolin-2-yl)benzenesulfonamide (16, C_25_H_22_ClN_3_O_4_S_2_)

Starting from 0.775 g **4** to 0.430 g 2-aminobenzophenone for 5 h, the reaction mixture was evaporated under the diminished pressure and the residue was crystallized from 2 cm^3^ acetonitrile. Solid was filtered out. The filtrate was evaporated to dryness and crystallized from 2 cm^3^ ethanol giving 0.350 g (33%) **16**. M.p.: 162–165 °C; TLC: *R*_f_ = 0.69 (benzene-EtOH 4:1); ^1^H NMR (500 MHz, DMSO-*d*_*6*_): *δ* = 0.92 (t, 3H, CH_3_), 2.24 (s, 3H, CH_3_), 3.83 (q, 2H, CH_2_), 3.97 (s, 2H, SCH_2_), 7.38–7.44 (m, 4H, arom), 7.54–7.57 (m, 2H, arom), 7.60–7.67 (m, 2H, arom), 7.86 (d, 1H, arom), 7.91 (t, 1H, arom), 7.97 (br s, 1H, arom), 13.65 (br s, 1H, SO_2_NH) ppm; ^13^C NMR (125 MHz, DMSO-*d*_*6*_/TFA): *δ* = 14.0, 19.1, 34.4, 61.2, 112.0, 117.0, 120.3, 125.3, 127.6, 128.4, 128.8, 130.2, 131.3, 132.4, 133.7, 134.3, 135.9, 136.4, 137.2, 139.4, 145.3, 153.3, 169.2 ppm; IR (KBr): $\bar{\nu }$ = 3244 (NH), 2978 (CH), 1723 (C=O), 1620 (NH def), 1567, 1514, 1493 (C=C, C=N), 1357, 1139 (SO_2_) cm^−1^; HRMS (ESI-TOF): *m/z* calcd. for C_25_H_22_ClN_3_O_4_S_2_ ([M+H]^+^) 528.0819, found 528.0717.

#### 2-Benzylthio-4-chloro-5-phenylcarbamoyl-*N*-(4-methylquinazolin-2-yl)benzenesulfonamide (17, C_29_H_23_ClN_4_O_3_S_2_)

Starting from 0.990 g **5** to 0.299 g 2′-aminoacetophenone for 5 h, the crude product was obtained. Extraction with 12 cm^3^ boiling ethanol gave 0.490 g (43%) **17**. M.p.: 228–230 °C; TLC: *R*_f_ = 0.70 (benzene-EtOH 4:1); ^1^H NMR (500 MHz, DMSO-*d*_*6*_): *δ* = 2.59 (s, 3H, CH_3_), 4.36 (s, 2H, CH_2_), 7.10–7.16 (m, 4H, arom), 7.34 (d, 2H, arom), 7.39 (t, 2H, arom), 7.46–7.51 (m, 2H, arom), 7.58 (s, 1H, arom), 7.73 (d, 2H, arom), 7.88 (t, 1H, arom), 8.08 (d, 1H, arom), 8.22 (br s, 1H, arom), 10.60 (s, 1H, NHCO), 13.46 (br s, 1H, SO_2_NH) ppm; ^13^C NMR (125 MHz, DMSO-*d*_*6*_/TFA): *δ* = 22.3, 36.0, 118.0, 119.3, 120.2, 124.3, 125.0, 127.2, 127.3, 127.5, 128.6, 129.2, 129.3, 132.2, 132.3, 133.5, 136.4, 136.5, 138.7, 139.2, 140.5, 153.2, 164.3 ppm; IR (KBr): $\bar{\nu }$ = 3417, 3318 (NH), 2968 (CH), 1673 (C=O), 1626 (NH def), 1585, 1526, 1497 (C=C, C=N), 1360, 1143 (SO_2_) cm^−1^; HRMS (ESI-TOF): *m/z* calcd. for C_29_H_23_ClN_4_O_3_S_2_ ([M+H]^+^) 575.0978, found 575.0973.

#### 2-Benzylthio-4-chloro-5-phenylcarbamoyl-*N*-(4-phenylquinazolin-2-yl)benzenesulfonamide (18, C_34_H_25_ClN_4_O_3_S_2_)

Starting from 0.990 g **5** to 0.430 g 2-aminobenzophenone for 3 h 45 min, the crude product was obtained. Extraction of byproducts with 7 cm^3^ boiling ethanol allowed to obtain 0.450 g (36%) **18**. M.p.: 243–245 °C; TLC: *R*_f_ = 0.70 (benzene-EtOH 4:1); ^1^H NMR (500 MHz, DMSO-*d*_*6*_): *δ* = 4.39 (s, 2H, CH_2_), 6.98–7.06 (m, 3H, arom), 7.16 (t, 1H, arom), 7.24–7.36 (m, 3H, arom), 7.40 (t, 2H, arom), 7.46–7.58 (m, 4H, arom), 7.60–7.68 (m, 2H, arom), 7.73 (d, 2H, arom), 7.87–7.97 (m, 2H, arom), 8.15 (br s, 1H, arom), 10.57 (s, 1H, NHCO), 13.70 (br s, 1H, SO_2_NH) ppm; ^13^C NMR (125 MHz, DMSO-*d*_*6*_/TFA): *δ* = 36.1, 117.0, 120.1, 124.2, 125.2, 127.4, 127.9, 128.4, 128.5, 128.9, 129.0, 129.1, 130.2, 131.2, 131.5, 132.3, 134.2, 135.8, 136.2, 136.3, 138.8, 139.2, 140.4, 153.3, 163.9 ppm; IR (KBr): $\bar{\nu }$ = 3396, 3290 (NH), 2926 (CH), 1660 (C=O), 1621 (NH def), 1582, 1551, 1494 (C=C, C=N), 1316, 1146 (SO_2_) cm^−1^; HRMS (ESI-TOF): *m/z* calcd. for C_34_H_25_ClN_4_O_3_S_2_ ([M+H]^+^) 637.1135, found 637.1133.

#### 2-Carbamoylmethylthio-4-chloro-5-(4-chlorophenylcarbamoyl)-*N*-(4-methylquinazolin-2-yl)benzenesulfonamide (19, C_24_H_19_Cl_2_N_5_O_4_S_2_)

Starting from 0.995 g **6** to 0.299 g 2′-aminoacetophenone for 5 h 15 min, the crude product was obtained. Extraction with 250 cm^3^ boiling ethanol gave 0.690 g (61%) **19**. M.p.: 288–292 °C (dec.); TLC: *R*_f_ = 0.42 (benzene-EtOH 4:1); ^1^H NMR (500 MHz, DMSO-*d*_*6*_): *δ* = 2.68 (s, 3H, CH_3_), 3.76 (s, 2H, CH_2_), 7.26 (s, 1H, CONH_A_), 7.45 (d, *J *= 8.3 Hz, 2H, 4-ClPh), 7.48–7.56 (m, 2H, arom), 7.58–7.62 (m, 2H, arom, CONH_B_), 7.76 (d, *J *= 8.3 Hz, 2H, 4-ClPh), 7.87 (br s, 1H, arom), 8.09 (d, 1H, arom), 8.26 (br s, 1H, arom), 10.77 (s, 1H, NHCO), 13.48 (s, 1H, SO_2_NH) ppm; ^13^C NMR (125 MHz, DMSO-*d*_*6*_/TFA): *δ* = 22.4, 36.1, 118.1, 119.3, 121.6, 125.0, 127.1, 128.1, 129.1, 132.0, 132.2, 133.6, 136.4, 138.0, 138.5, 141.1, 142.5, 153.4, 164.4, 169.7 ppm; IR (KBr): $\bar{\nu }$ = 3436, 3291, 3159 (NH), 2995, 2913, 2678 (CH), 1657, 1641 (C=O), 1591, 1512 (C=C, C=N), 1310, 1143 (SO_2_) cm^−1^; HRMS (ESI-TOF): *m/z* calcd. for C_24_H_19_Cl_2_N_5_O_4_S_2_ ([M+H]^+^) 576.0334, found 576.0340.

#### 2-Carbamoylmethylthio-4-chloro-5-(4-chlorophenylcarbamoyl)-*N*-(4-phenylquinazolin-2-yl)benzenesulfonamide (20, C_29_H_21_Cl_2_N_5_O_4_S_2_)

Starting from 0.995 g **6** to 0.430 g 2-aminobenzophenone for 4 h 45 min, the crude product was obtained. Extraction with 12 cm^3^ boiling ethanol gave 0.550 g (43%) **20**. M.p.: 310–313 °C (dec.); TLC: *R*_f_ = 0.52 (benzene-EtOH 4:1); ^1^H NMR (500 MHz, DMSO-*d*_*6*_): *δ* = 3.80 (s, 2H, CH_2_), 7.26–7.36 (m, 2H, arom, CONH_A_), 7.47–7.52 (m, 7H, arom), 7.58–7.61 (m, 3H, arom, CONH_B_), 7.77 (d, *J *= 8.3 Hz, 2H, 4-ClPh), 7.90–7.92 (m, 2H, arom), 8.19 (br s, 1H), 10.71 (s, 1H, NHCO), 13.75 (br s, 1H, SO_2_NH) ppm; ^13^C NMR (125 MHz, DMSO-*d*_*6*_/TFA): *δ* = 36.2, 117.0, 121.5, 125.3, 127.6, 128.1, 128.5, 128.9, 129.0, 130.3, 131.3, 131.5, 132.0, 134.2, 135.9, 136.4, 138.1, 138.6, 141.2, 153.5, 163.9, 169.6 ppm; IR (KBr): $\bar{\nu }$ = 3425, 3286 (NH), 2925 (CH), 1660, 1645 (C=O), 1582, 1556, 1493 (C=C, C=N), 1364, 1160 (SO_2_) cm^−1^; HRMS (ESI-TOF): *m/z* calcd. for C_29_H_21_Cl_2_N_5_O_4_S_2_ ([M+H]^+^) 638.0490, found 638.0489.

#### 2-Benzylthio-4-chloro-5-(4-chlorophenylcarbamoyl)-*N*-(4-methylquinazolin-2-yl)benzenesulfonamide (21, C_29_H_22_Cl_2_N_4_O_3_S_2_)

Starting from 1.060 g **7** to 0.299 g 2′-aminoacetophenone for 5 h, the crude product was obtained. Crystallization from 7 cm^3^ ethanol gave 0.620 g (51%) **21**. M.p.: 278–281 °C; TLC: *R*_f_ = 0.67 (benzene-EtOH 4:1); ^1^H NMR (500 MHz, DMSO-*d*_*6*_): *δ* = 2.61 (s, 3H, CH_3_), 4.37 (s, 2H, CH_2_), 7.07–7.12 (m, 3H, arom), 7.32 (d, 2H, arom), 7.44–7.48 (m, 3H, arom), 7.51 (d, 1H, arom), 7.59 (s, 1H, H-3), 7.76 (d, 2H, arom), 7.88 (t, 1H, arom), 8.07 (d, 1H, arom), 8.25 (s, 1H, H-6), 10.75 (s, 1H, NHCO), 12.50 (br s,1H, SO_2_NH) ppm; IR (KBr): $\bar{\nu }$ = 3321 (NH), 2924 (CH), 1681 (C=O), 1634 (NH def), 1586, 1537, 1493 (C=C, C=N), 1310, 1145 (SO_2_) cm^−1^; HRMS (ESI-TOF): *m/z* calcd. for C_29_H_22_Cl_2_N_4_O_3_S_2_ ([M+H]^+^) 609.0589, found 609.0593.

#### 2-Benzylthio-4-chloro-5-(4-chlorophenylcarbamoyl)-*N*-(4-phenylquinazolin-2-yl)benzenesulfonamide (22, C_34_H_24_Cl_2_N_4_O_3_S_2_)

Starting from 1.060 g **7** to 0.430 g 2-aminobenzophenone for 5 h, the crude product was obtained. Extraction with 9 cm^3^ boiling ethanol gave 0.620 g (46%) **22**. M.p.: 283–286 °C (dec.); TLC: *R*_f_ = 0.72 (benzene-EtOH 4:1); ^1^H NMR (500 MHz, DMSO-*d*_*6*_): *δ* = 4.39 (s, 2H, CH_2_), 6.98–7.08 (m, 3H, arom), 7.23–7.34 (m, 4H, arom), 7.46 (d, *J *= 8.8 Hz, 2H, 4-ClPh), 7.50–7.55 (m, 4H, arom), 7.60–7.68 (m, 2H, arom), 7.76 (d, *J *= 8.8 Hz, 2H, 4-ClPh), 7.90 (d, 1H, arom), 7.94–7.99 (m, 1H, arom), 8.13 (br s, 1H), 10.70 (s, 1H, NHCO), 13.75 (br s, 1H, SO_2_NH) ppm; ^13^C NMR (125 MHz, DMSO-*d*_*6*_/TFA): *δ* = 36.1, 117.0, 121.5, 125.2, 127.3, 127.9, 128.1, 128.4, 128.5, 128.8, 128.9, 129.1, 130.1, 131.2, 131.5, 131.9, 134.1, 135.7, 136.2, 136.3, 138.1, 138.9, 140.6, 145.0, 153.3, 163.9 ppm; IR (KBr): $\bar{\nu }$ = 3394, 3275 (NH), 2927, 2853 (CH), 1657 (C=O), 1622 (NH def), 1582, 1554, 1493 (C=C, C=N), 1309, 1147 (SO_2_) cm^−1^; HRMS (ESI-TOF): *m/z* calcd. for C_34_H_24_Cl_2_N_4_O_3_S_2_ ([M+H]^+^) 671.0745, found 671.0746.

#### 2-Benzylthio-4-chloro-5-(4-methylphenylcarbamoyl)-*N*-(4-methylquinazolin-2-yl)benzenesulfonamide (23, C_30_H_25_ClN_4_O_3_S_2_)

Starting from 1.020 g **8** to 0.299 g 2′-aminoacetophenone for 5 h, the crude product was obtained. Extraction of byproducts with 4 cm^3^ boiling ethanol gave 0.630 g (54%) **23**. M.p.: 273–277 °C (dec.); TLC: *R*_f_ = 0.68 (benzene-EtOH 4:1); ^1^H NMR (500 MHz, DMSO-*d*_*6*_): *δ* = 2.30 (s, 3H, CH_3_), 2.62 (s, 3H, CH_3_), 4.36 (s, 2H, SCH_2_), 7.06–7.12 (m, 3H, arom), 7.19 (d, *J *= 8.3 Hz, 2H, 4-MePh), 7.35 (d, 2H, arom), 7.46 (t, 1H, arom), 7.51 (d, 1H, arom), 7.58 (s, 1H, H-3), 7.61 (d, *J *= 8.3 Hz, 2H, 4-MePh), 7.89 (t, 1H, arom), 8.08 (d, 1H, arom), 8.24 (s, 1H, H-6), 10.51 (s, 1H, NHCO) ppm; IR (KBr): $\bar{\nu }$ = 3326 (NH), 2921 (CH), 1700 (C=O), 1670 (NH def), 1633, 1587, 1540 (C=C, C=N), 1316, 1150 (SO_2_) cm^−1^; HRMS (ESI-TOF): *m/z* calcd. for C_30_H_25_ClN_4_O_3_S_2_ ([M+H]^+^) 589.1135, found 589.1132.

#### 2-Benzylthio-4-chloro-5-(4-methylphenylcarbamoyl)-*N*-(4-phenylquinazolin-2-yl)benzenesulfonamide (24, C_35_H_27_ClN_4_O_3_S_2_)

Starting from 1.020 g **8** to 0.430 g 2-aminobenzophenone for 3.5 h, the crude product was obtained. Extraction of byproducts with 7 cm^3^ boiling ethanol gave 0.610 (47%) **24**. M.p.: 257–260 °C (dec.); TLC: *R*_f_ = 0.70 (benzene-EtOH 4:1); ^1^H NMR (500 MHz, DMSO-*d*_*6*_): *δ* = 2.31 (s, 3H, CH_3_), 4.38 (s, 2H, CH_2_), 6.98–7.08 (m, 3H, arom), 7.20 (d, *J *= 8.3 Hz, 2H, 4-MePh), 7.27–7.33 (m, 3H, arom), 7.48–7.54 (m, 5H, arom), 7.62 (d, *J *= 8.3 Hz, 2H, 4-MePh), 7.62–7.67 (m, 2H, arom), 7.90–7.95 (m, 2H, arom), 8.11 (br s, 1H, H-6), 10.48 (s, 1H, NHCO), 13.7 (br s, 1H, SO_2_NH) ppm; ^13^C NMR (125 MHz, DMSO-*d*_*6*_/TFA): *δ* = 20.1, 36.5, 117.3, 120.3, 120.7, 125.0, 127.3, 128.0, 128.2, 128.3, 128.7, 129.0, 129.2, 130.0, 130.9, 131.6, 132.6, 133.6, 134.3, 135.8, 136.0, 136.4, 138.8, 140.0, 145.7, 153.3, 163.6 ppm; IR (KBr): $\bar{\nu }$ = 3394, 3276 (NH), 2922 (CH), 1656 (C=O), 1621 (NH def), 1582, 1519, 1494 (C=C, C=N), 1362, 1147 (SO_2_) cm^−1^; HRMS (ESI-TOF): *m/z* calcd. for C_35_H_27_ClN_4_O_3_S_2_ ([M+H]^+^) 651.1291, found 651.1103.

### Cell culture and cell viability assay

All chemicals, if not stated otherwise, were obtained from Sigma-Aldrich (St. Louis, MO, USA). The MCF-7 and HeLa cell lines were purchased from Cell Lines Services (Eppelheim, Germany), the HCT-116 cell line was purchased from ATCC (ATCC-No: CCL-247). Cells were cultured in in Dulbecco’s modified Eagle’s medium (DMEM) supplemented with 10% fetal bovine serum, 2 mM glutamine, 100 units/cm^3^ penicillin, and 100 μg/cm^3^ streptomycin. Cultures were maintained in a humidified atmosphere with 5% CO_2_ at 37 °C in an incubator (Heraceus, HeraCell).

Cell viability was determined using the MTT (3-(4,5-dimethylthiazol-2-yl)-2,5-diphenyltetrazolium bromide) assay. Cells were seeded in 96-well plates at a density of 5 × 10^3^ cells/well and treated for 72 h with the examined compounds in the concentration range 1–100 μM (1, 10, 25, 50, and 100 μM). Following treatment, MTT (0.5 mg/cm^3^) was added to the medium and cells were further incubated for 2 h at 37 °C. Cells were lysed with DMSO and the absorbance of the formazan solution was measured at 550 nm with a plate reader (Victor, 1420 multilabel counter). The optical density of the formazan solution was measured at 550 nm with a plate reader (Victor, 1420 multilabel counter). The experiment was performed in triplicate. Values are expressed as the mean ± SD of at least three independent experiments.

### NCI screening

Cytotoxicity evaluation of compounds **9**–**14** and **17**–**24** was performed at the National Cancer Institute according to NCI-60 DTP human tumor cell line screen procedure [53–56].

### QSAR study

Three-dimensional models of studied compounds were built in Gaussian (Gaussian Inc.) software using DFT geometry optimization and B3LYP/6-31G(d) basis set. The optimized structures were imported to Dragon software (Talete, Milano, Italy) to calculate molecular descriptors. Descriptors with constant values or variance less than 0.0001 were discarded. Multiple linear regression along with forward stepwise algorithm and model validation was performed in Statistica software (Statsoft, Tulsa, USA). PLS, OPLS model and its leave one out cross-validation as well as VIP calculations were made in SIMCA (Umetrics, Umea, Sweden).

### Metabolic stability

Stock solutions of studied compounds were prepared at concentration of 10 mM in DMSO. Working solutions were prepared daily by dilution of stock with reaction buffer or acetonitrile, final concentration of organic solvent did not exceed 1%. Incubation mixture contained 10 μM of a studied derivative, 1 mM of NADPH (Sigma-Aldrich) and 0.5 mg/cm^3^ of pooled human liver microsomes (HLM, Sigma-Aldrich) in potassium phosphate buffer (0.1 M, pH 7.4). Incubation was carried out in thermostat at 37 °C and started by addition of studied compound. 50 mm^3^ samples were taken at starting point and after 5, 15, 30, 45, and 60 min. Enzymatic reaction was terminated by the addition of the equal volume of ice-cold acetonitrile. Incubations were performed in triplicates, the average *t*_1/2_ and standard deviation was calculated. Control incubations were performed without NADPH to assess chemical instability. After collection, samples were immediately centrifuged (10 min, 10,000 rpm) and resulted supernatant was directly analyzed or kept in − 80 °C until LC–MS analysis. Natural logarithm of a compound over IS peak area ratio was plotted versus incubation time. Metabolic half-time (*t*_1/2_) was calculated from the slope of the linear regression.

LC–MS analysis was performed on an Agilent 1260 system coupled to SingleQuad 6120 mass spectrometer (Agilent Technologies, Santa Clara, CA, USA). Poroshell EC-C18 (2.1 mm × 150 mm, 2.7 μm, Agilent Technologies, Santa Clara, CA, USA) was used in reversed-phase mode with gradient elution starting with 5% of phase A (10 mM ammonium formate in water) and 95% of phase B (10 mM ammonium formate in acetonitrile–water mixture, 95:5 v/v). The amount of phase B was linearly increased to 100% in 10 min. Total analysis time was 15 min at 40 °C, flow rate was 1 cm^3^/min and the injection volume was 5 mm^3^. The mass spectrometer was equipped with electrospray ion source and operated in positive ionization. Mass analyzer was set individually to each compound to detect [M+H]^+^ protonated molecule. MSD parameters of the ESI source were as follows: nebulizer pressure 35 psig (N_2_), drying gas 10 cm^3^/min (N_2_), drying gas temperature 300 °C, capillary voltage 3 kV, fragmentor voltage 150 V.

### In silico prediction of ADMET properties

Pharmacokinetic and toxicity properties (ADMET) of compounds were determined by an ADMET descriptors algorithm protocol of pkCSM approach (http://biosig.unimelb.edu.au/pkcsm/prediction) [44].
